# Nigeria’s Medical Exodus: Urgent Reforms to Retain Doctors

**DOI:** 10.7759/cureus.96421

**Published:** 2025-11-09

**Authors:** Badir Zakir

**Affiliations:** 1 Surgery, NES Healthcare, Leicester, GBR

**Keywords:** brain drain, healthcare policy and management, healthcare system, health system strengthening, medical workforce, nigeria, physician migration, retention strategies, working conditions

## Abstract

Nigeria is facing a worsening healthcare workforce crisis driven by the large-scale migration of its doctors, a phenomenon often described as ‘brain drain’. A 15-year cohort study found that nearly half of Nigerian medical graduates had emigrated within 15 years of qualification, contributing to a physician density of only about 3.8 per 10,000 people, far below global benchmarks for adequate physician coverage. Key factors include excessive and unregulated working hours, low and irregular remuneration, limited professional development, poor infrastructure, and weak institutional support. This exodus has resulted in understaffed hospitals, reduced mentorship and training capacity, impaired healthcare delivery, and widening inequities in access to care. Addressing this crisis requires coordinated reforms to establish and enforce reasonable duty-hour limits, improve remuneration, strengthen postgraduate training systems, and invest in leadership and infrastructure to promote doctor retention and sustain Nigeria’s health system.

## Editorial

Nigeria is experiencing an accelerating crisis in its healthcare workforce. Studies have documented a substantial outflow of Nigerian-trained doctors: a 15-year cohort study found that 48.9% of graduates from a major Nigerian medical school had migrated within 15 years of qualification, with the United Kingdom (48.5%), Canada (20.9%), and the United States (19.4%) as the main destinations [1]. According to the World Health Organization’s Global Health Observatory, Nigeria has approximately 3.8 doctors per 10,000 people, far below global benchmarks for adequate coverage [2]. With fewer than 1 doctor for every 2,600 people, even modest migration can severely disrupt already strained service delivery, particularly in rural and secondary hospitals where shortages are most acute [1,3].

One of the most critical drivers of this migration is the prevalence of unregulated and excessive working hours. Studies have shown that many resident doctors in Nigeria experience prolonged duty periods and high burnout risk, often linked to inadequate institutional regulation of work schedules [4,5]. Reports indicate that resident doctors in some tertiary hospitals have worked continuous on-call shifts ranging from 24 to 72 hours, with limited opportunities for rest, reflecting systemic gaps in duty-hour oversight and the absence of official national regulation of working hours [4-6]. While consecutive night shifts occur in many health systems, the absence of regulated limits or compensatory rest in Nigeria intensifies fatigue and compromises both physician well-being and patient safety, prompting many to emigrate as an act of self-preservation [4,5].

Financial constraints further exacerbate the problem. Despite rigorous medical training, Nigerian doctors often receive low and irregular salaries, with delayed or minimal hazard allowances, and face limited opportunities to supplement income due to institutional restrictions and heavy workloads [5,7]. In contrast, physicians in high-income countries, such as the United Kingdom, the United States, and Canada, earn substantially higher and more consistent remuneration, supported by pensions, housing allowances, and professional recognition [7,8]. This pronounced income and welfare disparity makes migration a rational pursuit of financial stability and professional fulfilment, mirroring broader economic migration trends across other skilled sectors but with uniquely severe consequences for healthcare delivery [5,7,8].

Career development challenges also play a significant role. Although postgraduate training pathways exist through the National Postgraduate Medical College of Nigeria and the West African Colleges, many trainees report dissatisfaction with the quality and structure of residency training, citing inadequate supervision, limited mentorship, and insufficient institutional backing [9]. In a multi-center survey of postgraduate trainers and trainees across accredited Nigerian institutions, over 70% rated the quality of training as fair or poor, and improving training quality abroad was identified as a leading reason for emigration [9]. These concerns are compounded by poor funding, inadequate infrastructure, and staffing constraints, which collectively hinder professional growth and long-term retention within the health system [4,10].

A cross-sectional survey of physicians across Nigeria by Onah et al. (2022) found that roughly 43% were actively seeking opportunities to emigrate, with poor remuneration, inadequate working conditions, insecurity, and limited career progression cited as primary drivers [11]. Hospitals consequently lose clinically experienced staff and struggle to maintain basic services as remaining clinicians absorb increasing caseloads [12,13]. Extremely high migration intention among doctors in training (about 70-74%) suggests that the problem is not only current attrition but also a compromised future pipeline [14]. Longitudinal data further demonstrate that nearly half of graduates emigrate within 15 years of qualification [1]: eroding institutional memory, weakening leadership succession, and diminishing supervision and mentorship necessary for training new doctors.

At the population level, the physician exodus threatens public-health resilience. The loss of doctors reduces capacity for maternal and child health services, weakens the management of infectious and chronic diseases, and limits readiness for public-health emergencies [5]. Lower physician density correlates with higher mortality in low- and middle-income settings, implying that continued reductions in doctor numbers are likely to translate into worse clinical outcomes, longer waits for care, delayed diagnoses, and higher preventable morbidity and mortality [15]. Hospital services experience interruptions, specialist units become understaffed or collapse, and research and mentorship activities decline as clinicians migrate [7].

The socioeconomic and equity consequences are equally profound. Migration deepens inequities in access, particularly for rural populations, increases workload and burnout among remaining staff, and imposes economic costs because public investment in medical training ultimately benefits other countries when graduates emigrate [16,17]. Brain drain also worsens patient-to-doctor ratios, lengthens wait times, reduces treatment outcomes, and increases mortality, undermining the performance of tertiary healthcare institutions [12,13]. Weak health system leadership further drives the crisis, as political neglect and poor governance fail to address the underlying causes of migration [18].

Collectively, available evidence suggests that doctor migration in Nigeria is more than a staffing problem. It disrupts training pipelines, depletes institutional leadership and specialist services, worsens population health indicators, and amplifies social and economic inequities. Addressing these consequences requires coordinated policies that retain clinicians through investment in infrastructure, institutional support, and effective workforce planning, while rebuilding systems for training, supervision, and leadership that sustain long-term health system resilience.

Addressing the well-documented challenges of excessive working hours and burnout requires urgent reforms that prioritize reasonable duty-hour limits, equitable remuneration, and stronger institutional leadership to enhance retention [4,5]. While existing postgraduate training pathways offer structured opportunities for professional growth, improving mentorship, supervision, and institutional support will make these systems more effective [9]. Retention strategies should integrate financial, professional, and psychosocial support, extending from the documented challenges of stress and burnout among doctors [4]. International collaboration, through strengthened training exchanges, capacity-building partnerships, leadership programs, and technical cooperation, can complement domestic reforms by enhancing institutional capacity, supporting workforce development, and fostering leadership [17]. Ultimately, sustainable retention will depend on consistent policy enforcement, improved working conditions, and a cultural shift that values and supports healthcare professionals, preventing continued workforce depletion and its far-reaching public-health consequences [12].

In conclusion, the emigration of Nigerian-trained doctors represents a critical threat to healthcare delivery, patient outcomes, and national development. To address this challenge, Nigeria should prioritize strengthening existing residency and fellowship frameworks, such as those of the National Postgraduate Medical College of Nigeria and the West African Colleges, by improving mentorship, supervision, and overall training quality [14]. Furthermore, integrating professional and psychosocial support systems that address burnout and promote well-being [4], alongside better regulation of working hours, enhanced remuneration, investment in infrastructure, and stronger institutional leadership, can help stabilize Nigeria’s medical workforce, strengthen the healthcare system, and safeguard the health of its population.
